# Genome protective effect of metformin as revealed by reduced level of constitutive DNA damage signaling

**DOI:** 10.18632/aging.100397

**Published:** 2011-10-28

**Authors:** H. Dorota Halicka, Hong Zhao, Jiangwei Li, Frank Traganos, Sufang Zhang, Marietta Lee, Zbigniew Darzynkiewicz

**Affiliations:** ^1^ Brander Cancer Research Institute and Department of Pathology, New York Medical College, Valhalla, NY 10595, USA; ^2^ Department of Biochemistry and Molecular Biology, New York Medical College, Valhalla, NY 10595, USA

**Keywords:** DNA replication stress, Reactive oxidant species (ROS), H2AX phosphorylation, ATM activation, cell cycle

## Abstract

We have shown before that constitutive DNA damage signaling represented by H2AX-Ser139 phosphorylation and ATM activation in untreated normal and tumor cells is a reporter of the persistent DNA replication stress induced by endogenous oxidants, the by-products of aerobic respiration. In the present study we observed that exposure of normal mitogenically stimulated lymphocytes or tumor cell lines A549, TK6 and A431 to metformin, the specific activator of 5'AMP-activated protein kinase (AMPK) and an inhibitor of mTOR signaling, resulted in attenuation of constitutive H2AX phosphorylation and ATM activation. The effects were metformin-concentration dependent and seen even at the pharmacologically pertinent 0.1 mM drug concentration. The data also show that intracellular levels of endogenous reactive oxidants able to oxidize 2',7'-dihydro-dichlorofluorescein diacetate was reduced in metformin-treated cells. Since persistent constitutive DNA replication stress, particularly when paralleled by mTOR signaling, is considered to be the major cause of aging, the present findings are consistent with the notion that metformin, by reducing both DNA replication stress and mTOR-signaling, slows down aging and/or cell senescence processes.

## INTRODUCTION

In live cells, DNA is continuously being damaged by reactive oxygen species (ROS), the by-products of aerobic respiration in mitochondria [1-6]. Exogenous oxidants originating from environmental pollutants [7],phagocyte-oxidative burst [8-10], and even iatrogenic factors [11], additionally contribute to DNA damage. Such DNA damage involves oxidation of the constituent DNA bases, particularly of guanine by formation of 8-oxo-7,8-dihydro-2'-deoxyguanosine (oxo8dG), base ring fragmentation, modification of deoxyglucose, crosslinking of DNA and protein, and induction of DNA double strand breaks (DSBs) [12, 13]. Another important injurious effect of endogenous and exogenous oxidants is peroxidation of lipids in cell membranes [14].

The extent of ROS-induced DNA damage varies widely in different studies [1-6]. According to one rather conservative estimate, about 5,000 DNA single-strand lesions (SSLs) are generated per nucleus during a single cell cycle of approximately 24 h duration [6]. About 1% of those lesions become converted to DSBs, mostly during DNA replication. This leads to formation of ~50 “endogenous DSBs”, the most severe and potentially mutagenic lesions [6]. DSBs can be repaired by two mechanisms, recombinatorial repair or nonhomologous DNA-end joining (NHEJ). The template-assisted recombinatorial repair is essentially error-free but takes place only when cells have already replicated their DNA which can serve as a template, namely in late-S and G_2_ phase of the cell cycle. DNA repair in cells lacking a template such as in G_1_ and early S phase occurs via the NHEJ mechanism. The latter is error-prone and may result in deletion of some base pairs [15, 16]. When such change occurs at the site of an oncogene or tumor suppressor gene it may promote carcinogenesis [17, 18]. It can also lead to translocations and telomere fusion, hallmarks of tumor cells [19]. The progressive accumulation of DNA damage with each sequential cell cycle has been considered to be the primary cause of cell aging and senescence [20]. However, the notion that persistent stimulation of mTOR-driven pathways (rather than the ROS-induced DNA damage) is the major mechanism responsible for aging appears to have more merit [21-27]. Oxidative DNA damage, on the other hand, by contributing to replication stress may be a factor enhancing the TOR-driven aging or senescence process [28].

Strategies for preventing cancer or slowing down aging are often directed at protecting DNA from oxidative damage. Protective agents can be identified by their ability to reduce formation of “endogenous DSBs”. The direct detection of endogenous DSBs in individual cells has been difficult because the leading methodology, single cell electrophoresis (comet) assay [29], lacks the desired sensitivity. The TUNEL assay, developed to label DSBs in apoptotic cells, also lack sufficient sensitivity [30, 31]. While the assays of DNA damage measurement in bulk offer greater sensitivity, these approaches do not allow one to relate the damage to individual cells, reveal any heterogeneity within cell populations, or the relationship of DSBs to cell cycle phase or apoptosis.

Among the early and most sensitive reporters of DNA damage, and in particular formation of DSBs, is the activation of the Ataxia Telangiectasia mutated protein kinase (ATM) through its autophosphorylation on Ser1981 [32], and the phosphorylation of histone H2AX on Ser139; the phosphorylated H2AX is designated as γH2AX [33]. Immunocytochemical detection of these events offers high sensitivity in assessment of DSBs formation in individual cells [34-37]. These biosensors of DNA damage have been used in conjunction with flow- or image-cytometry to assess DNA damage in cells exposed to a variety of exogenous genotoxins (reviews, [31, 38]). In fact, the high sensitivity of these biomarkers makes it possible to use them to detect and measure the extent of constitutive DNA damage induced by the metabolically generated ROS in untreated cells [39-41]. Furthermore, these markers can be used to explore the effectiveness of factors protecting nuclear DNA from endogenous oxidants [42-45]. Thus, the anti-oxidants (N-acetyl-L-cysteine, ascorbate, Celecoxib), inhibitors of glycolysis and oxidative phosphorylation (2-deoxy-D-glucose and 5-bromo-pyruvate), hypoxia (3-5% O_2_), confluency, low serum concentration, were all shown to distinctly reduce the level of constitutive ATM activation and H2AX phosphorylation [40-45]. Conversely, the factors enhancing metabolic activity (aerobic glycolysis) such as cell mitogenic activation, glucose, or dichloroacetate amplified the level of constitutive expression of γH2AX and activated ATM [42-45]. Collectively, these observations provide strong evidence that the extent of the ongoing DNA damage imposed by endogenous oxidants as well as the effectiveness of factors that protect from (or enhance) the damage can be assessed by analysis of the level of constitutive DNA damage signaling.

In the present study we tested whether metformin, a drug widely prescribed to treat type 2 diabetes, has the ability to modulate the level of constitutive DNA damage signaling. Metformin is a specific activator of 5'AMP-activated protein kinase (AMPK), a phylo-genetically conserved serine/threonine kinase that plays a key role in cellular energy homeostasis (reviews, [46-52]). AMPK is the energy sensor (“fuel gauge”) monitoring and regulating cellular energy in response to metabolic needs and nutritional environmental variations. This kinase is activated by low cellular energy status (increased AMP/ATP ratio) and responds by: (i) activating ATP-producing catabolic pathways such as glycolysis and fatty acids oxidation and (ii) suppressing the energy (ATP)-consuming anabolic pathways such as lipogenesis, gluconeogenesis and protein synthesis. Another effect of AMPK activation is inhibition of mammalian target of rapamycin (mTOR), the downstream effector of growth factor signaling pathways [51]. AMPK affects these activities by phosphorylating proteins regulating these pathways (instant effect) as well as by modulating transcription of genes encoding proteins of these pathways (delayed effect) [53-55]. AMPK itself is activated by the upstream mediator liver kinase B1 (LKB1) [52]. Activation of AMPK by metformin was shown to reduce intracellular reactive oxygen species (ROS) levels *via* upregulation of expression of the antioxidant thioredoxin through the AMPK-FOXO3 pathway [55].

There is a growing body of evidence that metformin may be considered a promising anti-aging candidate, applicable for life span extension, prevention and even treatment of cancer [22-27, 50, 56]. Given the above, it is of additional interest to know how metformin affects the level of constitutive DNA signaling in normal and tumor cells. Our present data show that in normal lymphocytes, as well as in cells of tumor lines the level of constitutive ATM activation and γH2AX expression was distinctly attenuated upon exposure to metformin. Also reduced was the level of intracellular ROS.

## RESULTS

**Figure 1:**
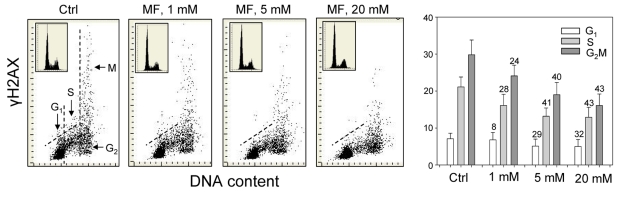
Effect of metformin (MF) on the level of constitutive γH2AX expression in A549 cells Exponentially growing A549 cells were left untreated (Ctrl) or treated with 1, 5 or 20 mM metformin for 48 h. Left panels present bivariate distributions of cellular DNA content *versus* intensity of γH2AX immunofluorescence (IF) detected with H2AX-Ser139 phospho-specific Ab in cells of these cultures; fluorescence of individual cells was measured by laser scanning cytometry (LSC) [76]. Based on differences in DNA content the cells were gated in G_1_, S and G_2_M phases of the cell cycle, as shown in the left panel, and the mean values of γH2AX IF for cells in each of these cell cycle phases by were obtained gating analysis. These mean values (+SD) are presented as the bar plots (right panel). The percent decrease in mean values of γH2AX expression of the metformin-treated cells with respect to the same phase of the cell cycle of the untreated cells is shown above the respective bars. The skewed dash line shows the upper level of γH2AX IF intensity for 97% of G_1_- and S- phase cells in Ctrl. The insets show cellular DNA content frequency histograms in the respective cultures.

The effect of metformin was tested on the level of constitutive expression of γH2AX and Ser1981-phoshorylated ATM in human lung adenocarcinoma A549 cells. The cells were grown attached on slides and the expression of these phospho-proteins was measured by laser scanning cytometry (LSC) [57]. The data provide clear evidence that expression of γH2AX in A549 cells growing in the presence of metformin for 48 h was reduced (Figure 1). The reduction was apparent at 1 mM, and was progressively more pronounced following exposure to 5 and 20 mM concentrations of metformin.

Across all the three metformin concentrations, the degree of reduction in γH2AX expression was more distinct in G_2_M- and S- phase cells compared to cells in the G_1_-phase of the cycle. The DNA content frequency histograms did not show major changes in the cell cycle distribution following 48 h treatment with up to 10 mM metformin, while only a modest decrease in the proportion of S-phase cells was apparent following exposure to 20mM metformin (Figure 1, insets).

**Figure 2:**
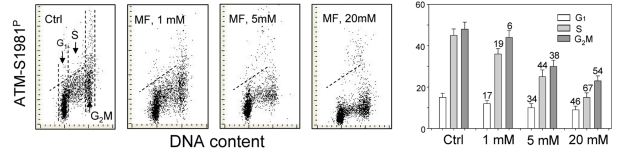
Effect of metformin (MF) on the level of constitutive ATM phosphorylation on Ser1981 in A549 cells Similar as in Figure 1, the cells were treated with 1, 5 or 20 mM MF for 48 h. Left panels present bivariate distributions of cellular DNA content vs intensity of ATM-S1981^P^ IF. The mean values of ATM-S1981^P^ for cells in G_1_, S, and G_2_M were obtained by gating analysis and are shown (+SD) as the bar plots (right panel). The skewed dash line shows the upper level of ATM-S198^P^ IF intensity for 97% of G_1_- and S- phase cells in Ctrl.

The effect of metformin on the level of constitutive expression of ATM phosphorylated on Ser1981 in A549 cells was strikingly similar to that of γH2AX (Figure 2). The degree of reduction of ATM-S1981^P^ was metformin-concentration dependent. While the decline in ATM activation was seen in all phases of the cell cycle, the most pronounced reduction was evident in S-phase cells (Figure 2).

**Figure 3:**
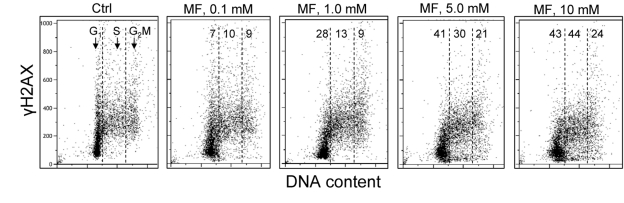
Effect of metformin on the level of constitutive expression of γH2AX in TK6 cells Exponentially growing TK6 cells were untreated (Ctrl) or were grown in the presence of 0.1, 1.0, 5.0 and 10 mM metformin (MF) for 48 h. The expression of γH2AX was detected with phospho-specific (Ser139-P) Ab and cell fluorescence was measured by flow cytometry. Based on differences in DNA content the cells were gated in G_1_, S and G_2_M phases of the cell cycle and the mean values of γH2AX IF for cells in each of these cell cycle phases were calculated. The numerical figures show the percent reduction in mean values of γH2AX IF of the metformin-treated cells with respect to the mean values of the untreated cells (Ctrl) in the respective phases of the cell cycle.

**Figure 4:**
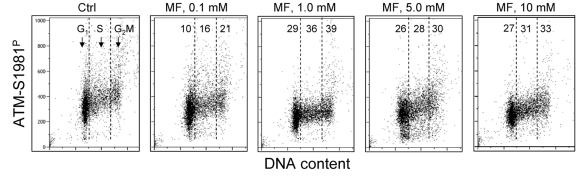
Effect of metformin on the level of constitutive expression of ATM-S1981^P^ Exponentially growing TK6 cells were untreated (Ctrl) or were grown in the presence of 0.1, 1.0, 5.0 and 10 mM metformin (MF) for 48 h. The expression of ATM-S1981^P^ was detected with phospho-specific Ab. As in Fig. 3, the cells were gated in G1, S and G2M phases of the cell cycle and the mean values of ATM-S1981^P^ for cells in each of these cell cycle phases were estimated. The figures show the percent reduction in mean values of ATM-S1981^P^ IF of the metformintreated cells with respect to the mean values of the untreated cells (Ctrl) in the respective phases of the cell cycle.

In the next set of experiments we have tested the effect of metformin on human lymphoblastoid TK6 cells. These cells grow in suspension and their fluorescence, upon staining with phospho-specific Abs, was measured by flow cytometry [57]. The data show that, similar to A549, the expression of γH2AX was also reduced in TK6 cells exposed to metformin (Figure 3). The effect could be seen (7 - 10% decrease) even at a metformin concentration as low as 0.1 mM, and was more pronounced (up to 44% reduction) at higher concentrations. In TK6 cells the reduction in γH2AX was more pronounced in G_1_ and S phase than in G_2_M phase cells. The level of constitutively activated ATM was also decreased in TK6 cells growing in the presence of metformin (Figure 4).

**Figure 5:**
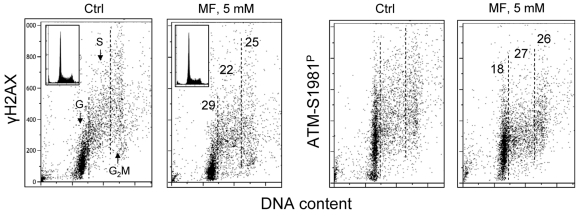
Effect of metformin on constitutive expression γH2AX and ATM-S1981^P^ in normal human proliferating lymphocytes Peripheral blood lymphocytes were mitogenically stimulated by phytohemagglutinin for 48 h and then were grown in the absence (Ctrl) or presence of 5 mM metformin (MF) for additional 24 h. The expression of γH2AX and ATM-S1981^P^ was detected with phospho-specific Abs and cell fluorescence we as measured by flow cytometry. The numerical figures show the percent reduction in expression of γH2AX and ATM-S1981^P^ of cells treated with metformin with respect to Ctrl, in the respective phases of the cell cycle.

Figure 5 illustrates the effect of metformin on proliferating human lymphocytes. The peripheral blood lymphocytes were stimulated to proliferate by the polyvalent mitogen phytohemagglutinin for 48 h and subsequently were grown in the absence or presence of 5 mM metformin for 24 h. The data show that, as was the case with the tumor cell lines A549 and TK6, growth of lymphocytes in the presence of 5mM metformin distinctly reduced both the level of constitutive expression of γH2AX as well as of ATM-S1981^P^.

**Figure 6:**
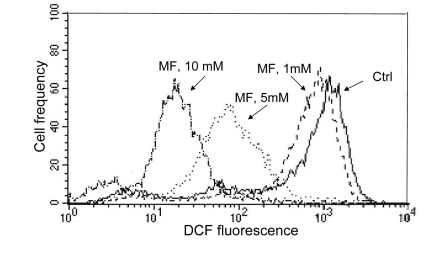
Effect of metformin on ability of TK6 cells to oxidize 2',7'-dihydro-dichlorofluorescein diacetate (H2DCF-DA) TK6 cells were untreated (Ctrl) or treated with 1, 5 or 10 mM metformin (MF) for 48 h. The cells were then incubated for 30 min with 10 μM H2DCF-DA and their fluorescence was measured by flow cytometry. While H2DCF-DA is not fluorescent, the product of its oxidation (DCF) by intracellular ROS shows strong green fluorescence. Note dramatic decline in fluorescence intensity of cells treated with 5 or 10 mM metformin.

As mentioned in the Introduction, the decline in the level of constitutive expression of γH2AX and phosphorylation of ATM was observed in cells treated with agents that decrease the level of endogenous oxidants such as ROS scavengers or antioxidants [39-45, 58]. Therefore, we assessed the effect of metformin on the abundance of reactive oxidants in human leukemic TK6 cells in the same cultures in which we observed the decline in expression of γH2AX (Figure 3) and ATM-S1981^P^ (Figure 4). As is quite evident from the data shown in Figure 6, the growth of TK6 cells for 48 h in the presence of metformin led to a decrease in the level of ROS that were detected by their ability to oxidize 2',7'-dihydro-dichlorofluorescein diacetate (H2DCF-DA); following oxidation by ROS the non-fluorescent substrate H2DCF-DA is converted to the highly fluorescent product DCF [59]. The effect was concentration dependent and the oxidation of H2DCF-DA was reduced by nearly two orders of magnitude at a 10 mM concentration of metformin compared to untreated cells (Figure 6).

## DISCUSSION

The present data demonstrate that exposure of either normal, mitogenically activated lymphocytes, or tumor cell lines (A549, TK6) to metformin leads to a decrease in the level of constitutive phosphorylation of H2AX on Ser139 and constitutive activation of ATM. The observed decrease was evident even at a concentration as low as 0.1 mM metformin (Figures 3 and 4). Pharmacokinetic data indicate that this concentration of metformin is of pharmacological relevance [60].Since the level of constitutive expression of γH2AX and ATM-S1981^P^ to a large extent reports DNA damage signaling in response to DNA damage by endogenous oxidants generated during aerobic respiration [39-45, 58]. the present findings would be consistent with a notion that metformin exerts protective effect on nuclear DNA against oxidative damage. These findings are consistent with the observation that exposure of cells to metformin lowered the extent of reactive oxidants that were able to oxidize the H2DCF-DA substrate (Figure 5). They are also in accordance with numerous studies in which a decrease in the level of ROS in cells treated with metformin has been observed [55, 61-65]. It appears that the mechanisms activated by metformin for neutralizing ROS such as upregulation of the antioxidant thioredoxin [55], and/or suppression of NAD(P)H oxidase activity [61] may prevail over the ROS-generating inhibitory effect on mitochondrial respiratory complex I or catabolic processes activated by AMPK [66, 67].

It should be noted that DNA damage signaling such as reported by H2AX phosphorylation and ATM activation do not necessarily indicate the actual DNA damage that involves formation of DNA strand breaks [68]. While some breaks may be formed during replication of DNA sections containing the primary oxidative lesions (e.g. oxo8dG) the presence of such lesions by themselves can induce persistent replication stress. The persistent replication stress combined with activation of mTOR pathways is considered to be the main mechanism contributing to aging and senescence [22-27, 69, 70]. Induction of replication stress by arrest in the cell cycle e.g. by upregulation of the CKI p21, with no evidence of actual DNA damage, elevates the level of constitutive DNA damage signaling (“pseudo-DNA damage response”) whereas attenuation of this “pseudo-DNA damage response” can be achieved by reduction in mTOR-signaling [29]. Likewise, the cell senescence induced by the replication stress triggered by low doses (1 – 2 nM) of the DNA damaging agent mitoxantrone, that is also accompanied by elevated levels of DNA damage signaling, was shown to be attenuated by the caloric restriction-mimicking drug 2-deoxy-D-glucose [71]. All this evidence collectively indicates that the observed constitutive DNA damage signaling occurs as a response to persistent DNA replication stress. Thus, by reducing the level of DNA damage signaling, as we presently see, metformin appears to alleviate the extent of the persistent DNA replication stress. Since metformin inhibits mTOR pathways, the reduction of replication stress by metformin may not only be mediated by attenuation of the oxidative stress through reduction of ROS, but also may be mediated by its direct inhibitory effect on mTOR [50-53].

**Figure 7:**
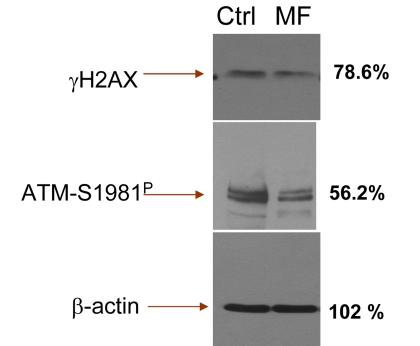
Detection of γH2AX and ATM-S1981^P^ in TK6 cells untreated (Ctrl) and treated with 5 mM metformin (MF) for 48 h, by immunoblotting The figures on right side of the blot represent the percent intensity of the scanned protein bands of the metformin-treated cells (UN-SCAN-IT gel 6.1) as that of the intensity of the respective protein bands of the untreated (Ctrl) cells.

Our observation that cells exposure to metformin reduces expression of γH2AX and ATM-S1981^P^ remains in contrast to recent data by Vazquez-Martin et al., that show the opposite, namely an activation of ATM and phosphorylation of H2AX in cells treated with metformin [72]. This report prompted us to repeat our experiments numerous times, using a variety of positive and negative controls. Yet in each experiment we observed that treatment of proliferating lymphocytes, TK6 or A549 cells led to a *decline* in expression of γH2AX and ATM-S1981^P^. We have also tested the A431 epidermoid carcinoma cells used by these authors [72]. The data show that treatment of A431with metformin *decreased* the level of H2AX and ATM phosphorylation (Supplemental data, Figure 1). To exclude the possibility of bias resulting from different methodologies we also assessed the effect of metformin on expression of γH2AX and ATM-S1981^P^ in TK6 cells using immonoblotting, the methodology used by the authors [72]. The results obtained by immunobloting (Figure 7) confirm all our immunocytochemical data (Figs. 1-5) by showing a distinct reduction of γH2AX and ATM-S1981^P^ in cells treated with metformin. In fact, the reduction in expression of ATM-S1981^P^ was nearly 45% related to the control. We have also observed that constitutive H2AX phosphorylation and ATM activation in quiescent A549 cells, maintained for 5 days at high cells density (>10^6^ cells/ml) with no medium change also was reduced by treatment with metformin (Supplemental data Figure 2). The effect of metformin, thus, was unrelated as to whether the cells were in exponential- or stationary- phase of growth. Our data also concur with the findings of Nilsson et al., who did not detect any induction of γH2AX in U2OS or HT1080 cells treated with 40 mM metformin [73]. Actually, careful inspection of their data provides some evidence of a decline in expression of γH2AX upon treatment with metformin [73]. At present we see no explanation for the apparent discrepancy of our results (and the data of Nilsson et al., [73]) versus the data presented by Vazquez-Martin al., [72].

As mentioned, cell aging and senescence appear to be driven by persistent mTOR activation in conjunction with DNA replication stress; the latter can be induced by ROS as well as by inhibition of cell cycle progression, such as activation of CKIs. DNA replication stress and mTOR activation are being reported by the elevated level of constitutive DNA damage signaling (“pseudo-DNA damage response”) [29, 71]. As shown in the present study, the effectiveness of potential anti-aging factors such as metformin may be tested by monitoring their effect on constitutive DNA damage signaling. This approach offers novel means to assess the anti-aging or aging-promoting properties of different factors suspected of such activities. Assessment of DNA damage signaling may serve to detect both genotoxicity [38, 74] as well as genome-protective mechanisms related to attenuation of DNA replication stress.

## MATERIALS AND METHODS

### Cells, cell treatment

Human lung carcinoma A549 cells, epidermoid carcinoma A431 and lymphoblastoid TK6 cells were obtained from American Type Culture Collection (ATCC CCL-185, Manassas,VA). Human peripheral blood lymphocytes were obtained by venipuncture from healthy volunteers and isolated by density gradient centrifugation. A549 cells were cultured in Ham's F12K, TK6 and lymphocytes were cultured in RPMI 1640 and A431cells in Dulbecco modified Eagle medium, with 2 mM L-glutamine adjusted to contain 1.5 g/L sodium bicarbonate supplemented with 10% fetal bovine serum (GIBCO/Invitrogen, Carlsbad, CA). Adherent A549 and A431 cells were grown in dual-chambered slides (Nunc Lab-Tek II), seeded with 10^5^ cells/ml suspended in 2 ml medium per chamber. TK6 cells and lymphocytes were grown in suspension; lymphocyte cultures were treated with the polyvalent mitogen phytohemaglutinin (Sigma /Aldrich; St Louis, MO) as described [75]. During treatment with metformin (1,1-dimethylbiguanide; Calbiochem, La Jolla, CA) the cells were in exponential phase of growth unless indicated otherwise. After exposure to metformin at various concentrations and for specified periods of time (as shown in figure legends) the cells were rinsed with phosphate buffered salt solution (PBS) and fixed in 1% methanol-free formaldehyde (Polysciences, Warrington, PA) for 15 min on ice The cells were then transferred to 70% ethanol and stored at −20 °C for up to 3 days until staining.

### Detection of H2AX phosphorylation and ATM activation

The cells were washed twice in PBS and with 0.1% Triton X-100 (Sigma) in PBS for 15 min and with a 1% (w/v) solution of bovine serum albumin (BSA; Sigma) in PBS for 30 min to suppress nonspecific antibody (Ab) binding. The cells were then incubated in 1% BSA containing a 1:300 dilution of phospho-specific (Ser139) γH2AX mAb (Biolegend, San Diego, CA or with a 1:100 dilution of phospho-specific (Ser1981) ATM mAb (Millipore, Tamecula, CA). The secondary Ab was tagged with AlexaFluor 488 fluorochrome (Invitrogen/Molecular Probes, used at 1:200 dilution). Cellular DNA was counterstained with 2.8 μg/ml 4,6-diamidino-2-phenylindole (DAPI; Sigma). Each experiment was performed with an IgG control in which cells were labeled only with the secondary AlexaFluor 488 Ab, without primary Ab incubation to estimate the extent of nonspecific adherence of the secondary Ab to the cells. The fixation, rinsing and labeling of A549 or A431 cell was carried out on slides, and lymphocytes and TK6 cells in suspension. Other details have been previously described [38-40].

### Analysis of cellular fluorescence

*A549 and A431 cells:* Cellular immunofluorescence representing the binding of the respective phospho-specific Abs as well as the blue emission of DAPI stained DNA was measured with an LSC (iCys; CompuCyte, Westwood, MA) utilizing standard filter settings; fluorescence was excited with 488-nm argon, helium-neon (633 nm) and violet (405 nm) lasers [76]. The intensities of maximal pixel and integrated fluorescence were measured and recorded for each cell. At least 3,000 cells were measured per sample. Gating analysis was carried out as described in Figure legends. *TK6 cells and lymphocytes:* Cellular fluorescence was measured by using a MoFlo XDP (Beckman-Coulter, Brea, CA) high speed flow cytometer/sorter. DAPI fluorescence was excited with the UV laser (355-nm) and AlexaFluor 488 with the argon ion (488-nm) laser.

### Protein immonoblotting

Nitrocellulose membrane was blocked with 5% w/v nonfat dry milk in TBST (20 mM TrisHCl, pH 7.4, 150 mM NaCl, 0.05% Tween 20) for 1h at room temperature. The blot was then incubated with the primary antibody either phospho-specific (Ser139) γH2AX mAb (Biolegend) or a phospho-specific (Ser1981) ATM mAb (Millipore) at 1:500 dilution overnight at 4 °C. After three washes in TBST, the blot was incubated with HRP-conjugated goat anti-mouse IgG (Pierce, Rockford, IL) for 1h at room temperature and washed with TBST three times. SuperSignal West Pico chemiluminescence substrate (Pierce) was used for signal production.

## SUPPLEMENTARY FIGURES

Supplemental Figure 1 Effect of metformin (MF) on the level of constitutive expression of γH2AX and ATM-S1981P in A431 cells Exponentially growing A431 cells were left untreated (Ctrl) or treated with 5 mM metformin for 48 h. γH2AX and ATM-S1981 immunofluorescence (IF) was detected with the phospho-specific Abs and cells fluorescence was measured by laser scanning cytometry.^75^ Based on differences in DNA content the cells were gated in G_1_, S and G_2_M phases of the cell cycle and the mean values of γH2AX and ATM-S1981^P^ IF for cells in each of these cell cycle phases by were obtained gating analysis. The percent reduction of these mean values of the metformin-treated related to the untreated (Ctrl) cells is shown in the respective panels (the means of the three separate bands per each protein). The insets show DNA content frequency histograms in the untreated and metformin-treated cultures.

Supplemental Figure 2 Effect of metformin (MF) on the level of expression of γH2AX in TK6 cells in stationary cultures TK6 cells were maintained at high cell density (>10^6^ cells/ml) with no medium change for 5 days, then cells were left untreated (Ctrl) or treated with 5 mM metformin for 24 h (MF). The percent decline in mean values of γH2AX IF of cells in G_1_, S, and G_2_M phases of the cycle in the metformin-treated culture is shown in the MF panel.
