# Supplementary figures and images for: Genome protective effect of metformin as revealed by reduced level of constitutive DNA damage signaling
Source: Aging (Albany NY). 2011 Oct 28;3(10):1028–38. doi: 10.18632/aging.100397 (PMC3229966; doi:10.18632/aging.100397)

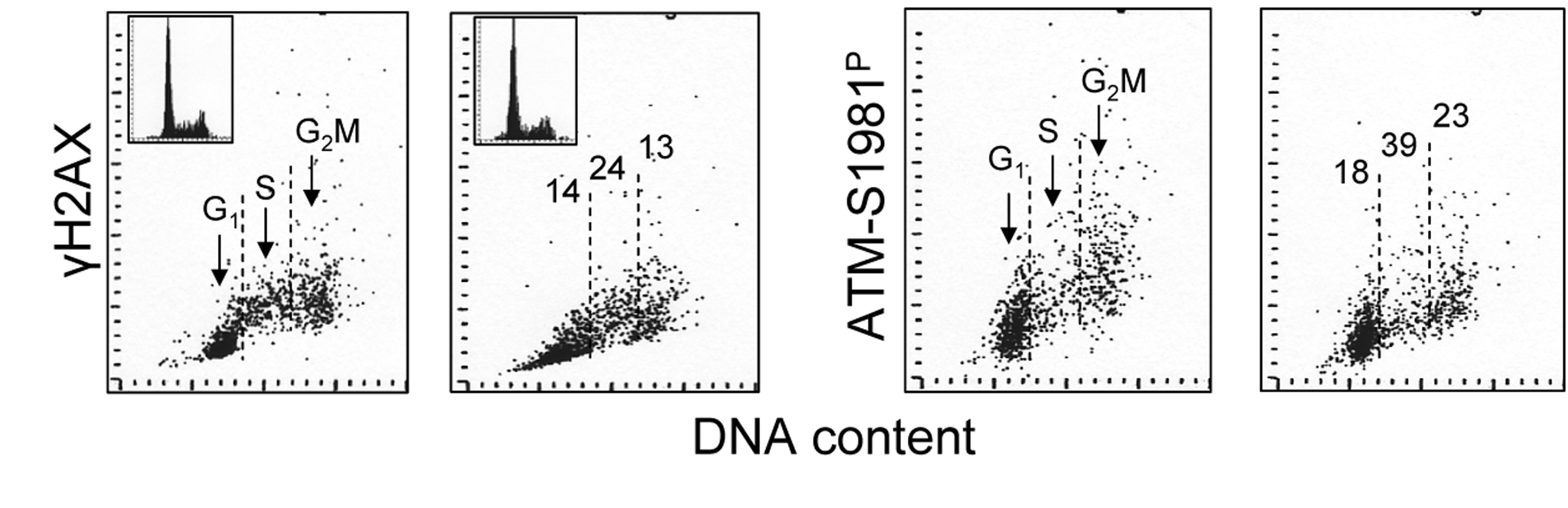

Supplement: Supplemental Figure 1 — Exponentially growing A431 cells were left untreated (Ctrl) or treated with 5 mM metformin for 48 h. γH2AX and ATM-S1981 immunofluorescence (IF) was detected with the phospho-specific Abs and cells fluorescence was measured by laser scanning cytometry.75 Based on differences in DNA content the cells were gated in G1, S and G2M phases of the cell cycle and the mean values of γH2AX and ATM-S1981P IF for cells in each of these cell cycle phases by were obtained gating analysis. The percent reduction of these mean values of the metformin-treated related to the untreated (Ctrl) cells is shown in the respective panels (the means of the three separate bands per each protein). The insets show DNA content frequency histograms in the untreated and metformin-treated cultures. [file aging-03-1028-s001.tif]

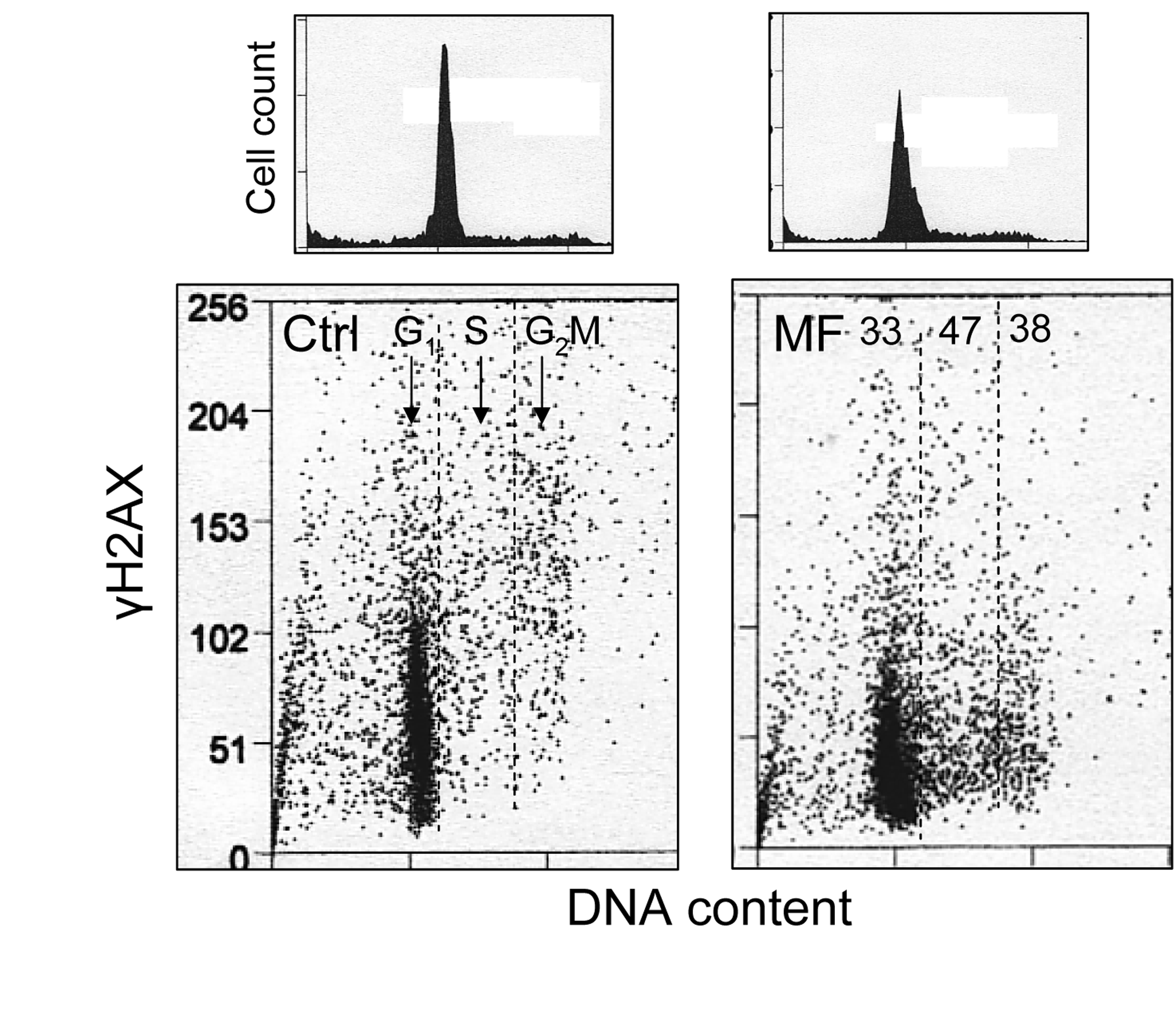

Supplement: Supplemental Figure 2 — TK6 cells were maintained at high cell density (>106 cells/ml) with no medium change for 5 days, then cells were left untreated (Ctrl) or treated with 5 mM metformin for 24 h (MF). The percent decline in mean values of γH2AX IF of cells in G1, S, and G2M phases of the cycle in the metformin-treated culture is shown in the MF panel. [file aging-03-1028-s002.tif]
